# Pooled analysis of Xpert Bladder Cancer based on the 5 mRNAs for rapid diagnosis of bladder carcinoma

**DOI:** 10.1186/s12957-021-02154-0

**Published:** 2021-02-09

**Authors:** Ye-Ling Liu, Xue-Lin Wang, Xiao-Hui Yang, Xiao-Huan Wu, Guo-Xin He, Li-Min Xie, Xun-Jie Cao, Xu-Guang Guo

**Affiliations:** 1 Department of Clinical Laboratory Medicine, The Third Affiliated Hospital of Guangzhou Medical University, Guangzhou, 510150 China; 2 Department of Clinical Medicine, The Third Clinical School of Guangzhou Medical University, Guangzhou, 511436 China; 3 Key Laboratory for Major Obstetric Diseases of Guangdong Province, The Third Affiliated Hospital of Guangzhou Medical University, Guangzhou, 510150 China; 4 Key Laboratory of Reproduction and Genetics of Guangdong Higher Education Institutes, The Third Affiliated Hospital of Guangzhou Medical University, Guangzhou, 510150 China

**Keywords:** Xpert Bladder Cancer, Bladder cancer, Cystoscopy

## Abstract

**Background:**

Xpert Bladder Cancer is a detection method developed in recent years, designed with the functions of integrating sample automatically, nucleic acid amplification, and target sequence detection. It is a urine assay targeting five mRNAs (CRH, IGF2, UPK1B, ANXA10, and ABL1). The purpose of this article is to review the accuracy of Xpert Bladder Cancer in the follow-up diagnosis of bladder cancer and evaluate the role of Xpert Bladder Cancer in detecting the recurrence of non-muscle-invasive bladder cancer in the round.

**Methods:**

In the database of Embase, PubMed, Web of Science, and Cochrane Library, the articles published up to October 13, 2020, were searched and screened based on the exclusion and inclusion criteria, and data were extracted from the included studies. The sensitivity, specificity, negative likelihood ratio, positive likelihood ratio summary of receiver operating characteristic curves, and diagnostic odds ratio were combined by the Meta-DiSc 1.4 software. The Stata 12.0 software was used to obtain the assessment of publication bias.

**Results:**

A total of 8 articles involving eight fourfold tables were finally identified. The pooled sensitivity and specificity of Xpert Bladder Cancer in the diagnosis of bladder cancer were 0.71 and 0.81, respectively. The positive likelihood ratio and negative likelihood ratio were 3.74 and 0.34, respectively. The area under the curve was 0.8407. The diagnostic odds ratio was 11.99. Deeks’ funnel plot asymmetry test manifested no publication bias.

**Conclusions:**

In summary, Xpert Bladder Cancer presents high accuracy and specificity in monitoring bladder cancer compared with cystoscopy. More researches are still required to further confirm this conclusion.

**Supplementary Information:**

The online version contains supplementary material available at 10.1186/s12957-021-02154-0.

## Introduction

Bladder cancer (BC), ranking the 12th among the most common malignant tumors worldwide and the second among the most common cancers in the genitourinary system, has been on the rise in popularity year by year [1, 2]. Invading urothelial histology principally, bladder cancer is categorized into muscle-invasive bladder cancer and non-muscle-invasive bladder cancer (NMIBC), which is a cancer of the urinary tract that only progresses to the mucosa or submucosa and accounts for 70–75% of all cases [3–6]. NMIBC can be classified into high-grade (HG) cancer and low-grade (LG) cancer according to the degree of malignant transformation [7]. In early treatment, transurethral resection (TUR) is the most frequently adopted method in patients with NMIBC [8]. However, patients with bladder cancer take a high risk of tumor progression and recurrence within 5 years of the initial treatment, making up 50 to 70% of all patients [3, 9]. Lifelong follow-up is recommended for patients with tumors with an intermediate or a high risk of recurrence or progression [10]. Whereas on a regular basis cystoscopy is the most widely adopted measure for NMIBC treatment and is regarded as the gold standard, it has to be conducted regularly and can be inconvenient and uncomfortable with patients due to its invasiveness [11]. Some patients develop pink urine or hematuria after cystoscopy, which can even be complicated by infection, leading to fever [12–14]. Cytological test where doctors often employ for follow-up is not perfect either in that it is good in specificity but not in sensitivity. What is more, the results of the cytological test are largely influenced by the observer [15]. Therefore, a non-invasive and highly efficient detection method is desperately needed to avoid follow-up cystoscopy and cytological test for the increasing population of non-muscle-invasive bladder cancer patients.

Based on the detection of five mRNA targets in urine (CRH, IGF2, UPK1B, ANXA10, and ABL1), Xpert Bladder Cancer (Xpert BC) is a recently developed detector for the detection of bladder cancer, which is non-invasive and highly economical [16, 17]. It can automatically complete the task of sample preparation, amplification of nucleic acid and detection of the target sequence, etc. [15]. Xpert BC assay provided a “negative” or “positive” result based on the results of a linear discriminant analysis (LDA), which depended on a regression algorithm that utilizes the cycle threshold (Ct) results of the five mRNA targets [5]. In recent years, researches on the diagnostic accuracy of Xpert BC in the follow-up of bladder cancer have been carried out in full swing. Pichler et al. for the first time reported Xpert BC Monitor and documented that even in LG and pTa diseases, it could monitor with high sensitivity [5]. Hurle et al. discovered that 33.4% of cystoscopy can be avoided if Xpert BC is applied [11]. One of the research of D’Elia et al. suggested that the sensitivity of the Xpert BC monitoring test was significantly higher than that of the cytological test, while its specificity failed to reach the cytological level [9]. Valenberg et al. pointed out that Xpert BC improved NPV in bladder cancer patients during follow-up compared with urology and cytology [15].

It has been fully documented that Xpert BC has high sensitivity and non-invasion in detecting the recurrence of NMIBC and is widely regarded as highly promising for future application. However, the differences between individual studies are a major impediment, and it still calls for a comprehensive assessment in terms of its diagnostic value [18, 19]. This study is aimed at reviewing the performance of Xpert BC in the follow-up of bladder cancer and evaluating the role of Xpert BC in detecting NMIBC recurrence in the round.

## Materials and methods

### Screening criteria

Included articles have to meet all the following criteria: (1) the samples used in the experiment are human samples; (2) English literature only; (3) the research object is bladder cancer; (4) diagnostic test method: Xpert BC and cystoscopy tests, the latter being the gold standard; and (5) the data extracted is sufficient to construct a 2 × 2 table.

Excluded documents meet one or more of the following criteria: (1) samples are animal samples or from other sources; (2) duplicate literature; (3) abstracts, lectures, conference records, and reviews; (4) articles from which the data extracted are not enough to make a 2 × 2 table; (5) lack of a gold standard for the diagnostic test method, or Xpert BC is not used for the test; and (6) patients with a history of bladder cancer or confirmed by cystoscopy.

### Search methods

The keywords “Xpert Bladder Cancer” and “bladder cancer,” together with their synonyms from EMTREE terms and Medical Subject Headings (MeSH), were used to search articles published in Embase, PubMed, Web of Science, and Cochrane Library until October 13, 2020. The reference list that seemed to meet the inclusion criteria was manually searched to determine whether it could be included. The collected literature was further screened according to the inclusion and exclusion criteria.

### Data extraction

After the articles were finally selected according to the abovementioned standard, the EndNote X9 software was employed respectively by the four researchers to collect data of these studies, including the authors’ name, year of publication, design of the study, LDA, type of cancer, sample size, gold standard, patient population, subject categories, true positive, false positive, true negative, and false negative, which were then recorded in an Excel form to construct a 2 × 2 table. Four researchers were divided into two groups, and the data were extracted independently by two researchers in each group. Should any discrepancy appear among the data or the opinions of the two researchers, a third-party researcher would review the article and make the decision after due discussion.

### Quality assessment

The Quality Assessment Standard for Diagnostic Accuracy Studies (QUADAS-2) was employed to evaluate the quality of the studies included, which contains eleven items and each of them is rated “yes,” “no,” or “unclear,” and the results were recorded in the form of quality evaluation. QUADAS-2 consists of four parts, i.e., reference standard, patient selection, flow and timing, and the index test, which is assessed to determine the risk of bias in the articles included [20]. All publications which are qualified are independently assessed by two reviewers. The differences were resolved through discussion and consultation with the third reviewer.

### Data analysis

The Meta-DiSc 1.4 software was employed for data analysis [21]. After analysis, diagnostic accuracy indicators with 95% confidence intervals (95% CIs) were obtained: sensitivity, specificity, positive likelihood ratio (PLR), negative likelihood ratio (NLR), diagnostic odds ratio (DOR), and summary of receiver operating characteristic curves (SROC). On the premise that the significant coefficient is set to *P* < 0.05, in order to study and evaluate the heterogeneity caused by the threshold effect, the Spearman correlation coefficient was used for analysis and testing, and heterogeneity was judged by Cochran’s *Q* and *I*^2^ tests. In addition, the Stata 12.0 software was used to produce a chart about publication bias to rule out possible errors caused by the tendency to publish studies with positive data.

## Results

### Literature retrieval process

After a systematic search, a total of 69 articles was obtained, including 11 from PubMed, 37 from Embase, 20 from the Web of Science, and 1 from the Cochrane Library. After excluding duplicates, 33 articles were retained. After reviewing the titles and abstracts, 12 articles were removed. At last, 8 remaining articles were subject to full-text screening. Finally, eight articles were included, from which data were extracted to construct eight fourfold tables for analysis [5, 7, 9, 11, 15, 17, 22, 23]. The detailed flow chart is shown in Additional file 1.

### Characteristics presented in the studies and methodological quality

Eight studies were published between 2017 and 2020, of which 2537 samples were included in the meta-analysis. Of the 8 articles, 3 were conducted from America, 3 were from Italy, 1 was from Egypt, and 1 was from Australia. All articles adopted the prospective experimental design, and the gold standard was mainly cystoscopy. The limit value of LDA of most articles was set to 0.4 or 0.5, and only one was set to − 20 to 20 [5]. Most of the patients in the study were characterized by a history of bladder cancer or a diagnosis of bladder cancer by cystoscopy. The characteristics of these studies were summarized in Table 1. Evaluation of the quality of the included articles was conducted (Table 2).

**Table 1:**
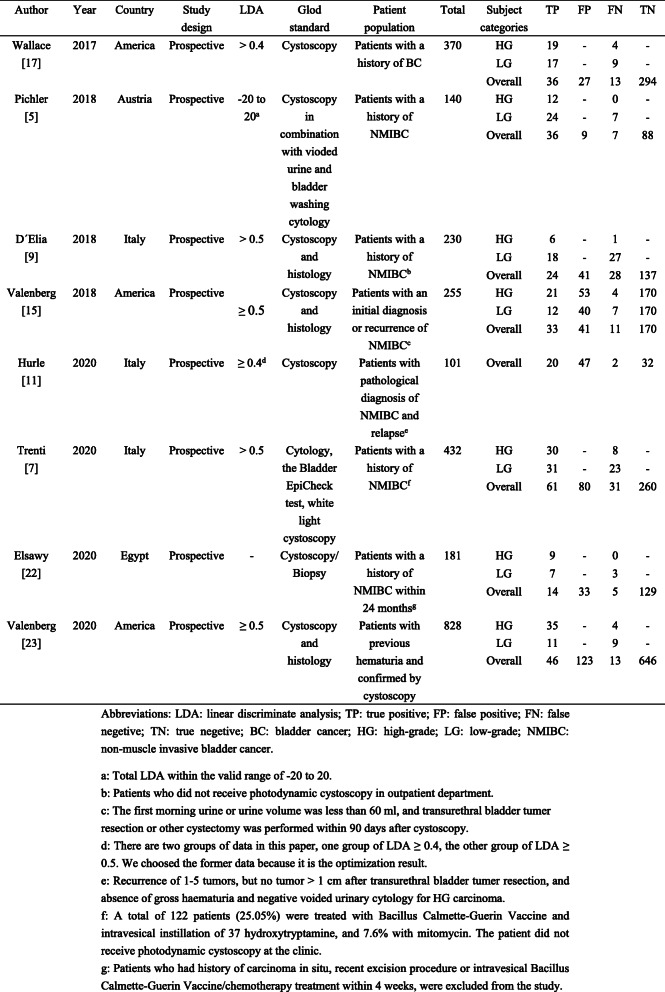
Characteristics of the included studies

**Table 2:**
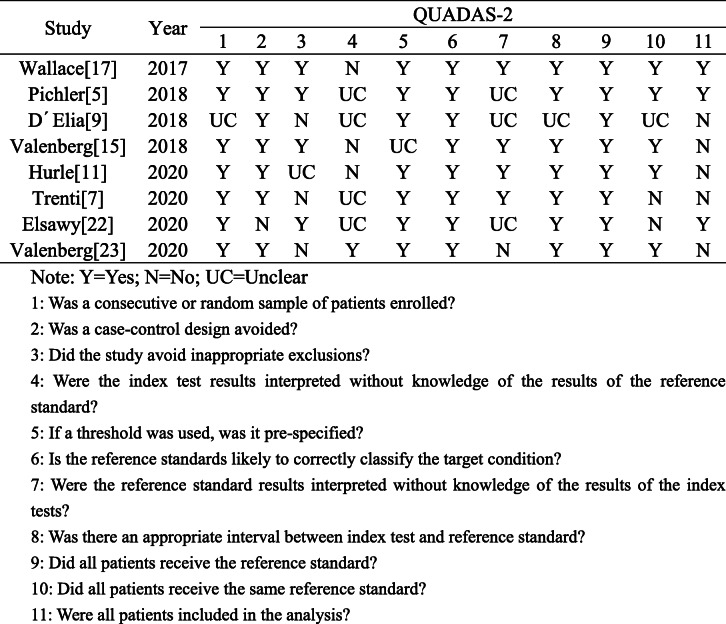
Quality assessment of the included studies

### Data analysis

**Fig. 1:**
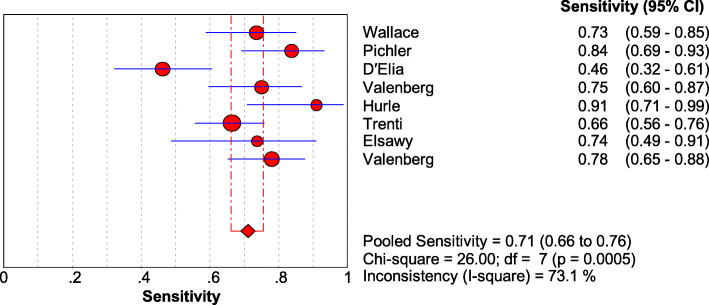
Forest plots for the combined sensitivity of Xpert Bladder Cancer

**Fig. 2:**
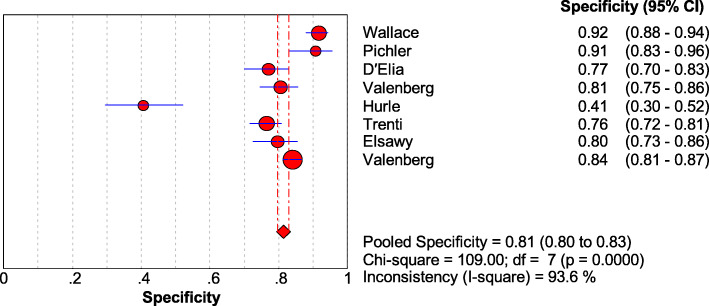
Forest plots for the combined specificity of Xpert Bladder Cancer

**Fig. 3:**
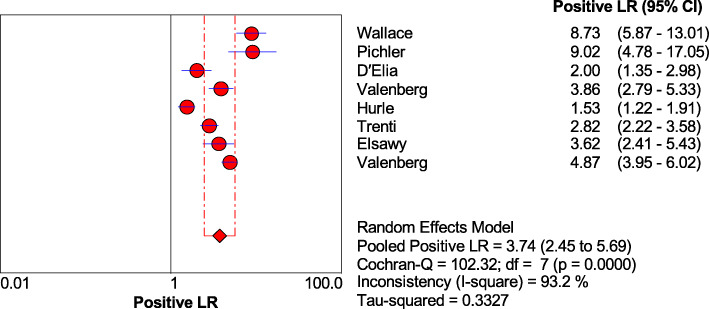
Forest plots for the combined positive LR of Xpert Bladder Cancer

**Fig. 4:**
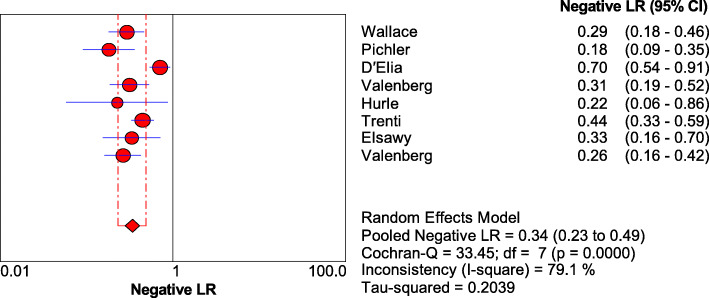
Forest plots for the combined negative LR of Xpert Bladder Cancer

**Fig. 5:**
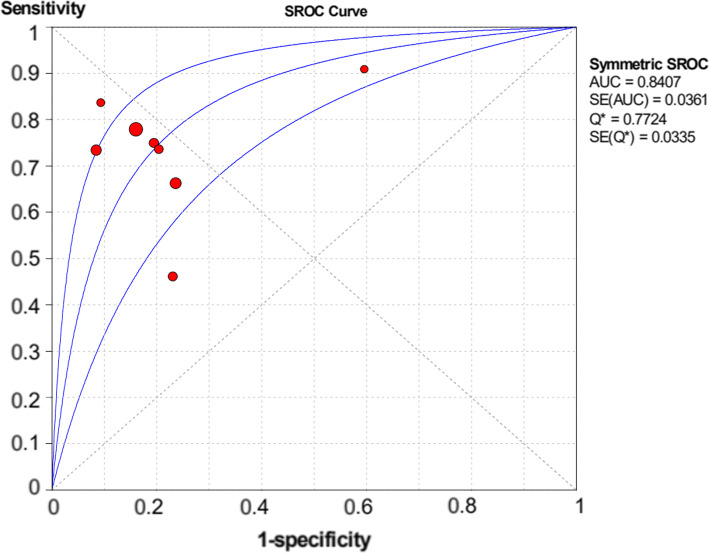
Summary of receiver operating characteristic curves of bladder cancer detected by for Xpert Bladder Cancer

As shown in Figs. 1, 2, 3, and 4, the sensitivity, specificity, PLR, and NLR of bladder cancer combination detected by Xpert BC were respectively 0.71 [95% CI (0.66~0.76)], 0.81 [95% CI (0.80~0.83)], 3.74 [95% CI (2.45~5.69)], and 0.34 [95% CI (0.23~0.49)]. As can be observed in Fig. 5, AUC = 0.8407, and the *Q* index was 0.7724 (SE = 0.0335).

### Heterogeneity analysis

**Fig. 6:**
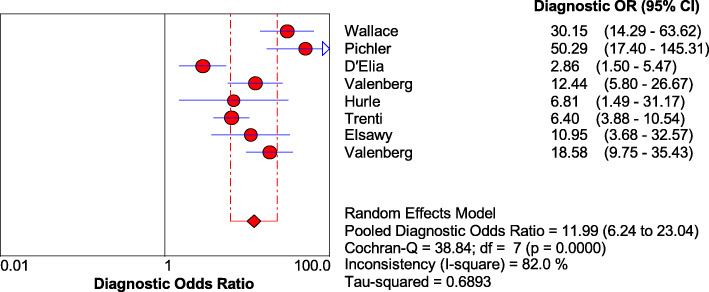
Forest plots for the combined diagnostic OR of Xpert Bladder Cancer

In the analysis of the threshold effect, the Spearman correlation coefficient was − 0.071, and *P* value was 0.867, which signified no threshold effect in the articles included. As shown in Fig. 6, the DOR is 11.99 [95% CI (6.24~23.04)], Cochran *Q* = 38.84 (*P* ≤ 0.001), and the inconsistency = 82.0% (inconsistency > 50%), which indicated low heterogeneity in the non-threshold effect.

### Assessing publication bias

**Fig. 7:**
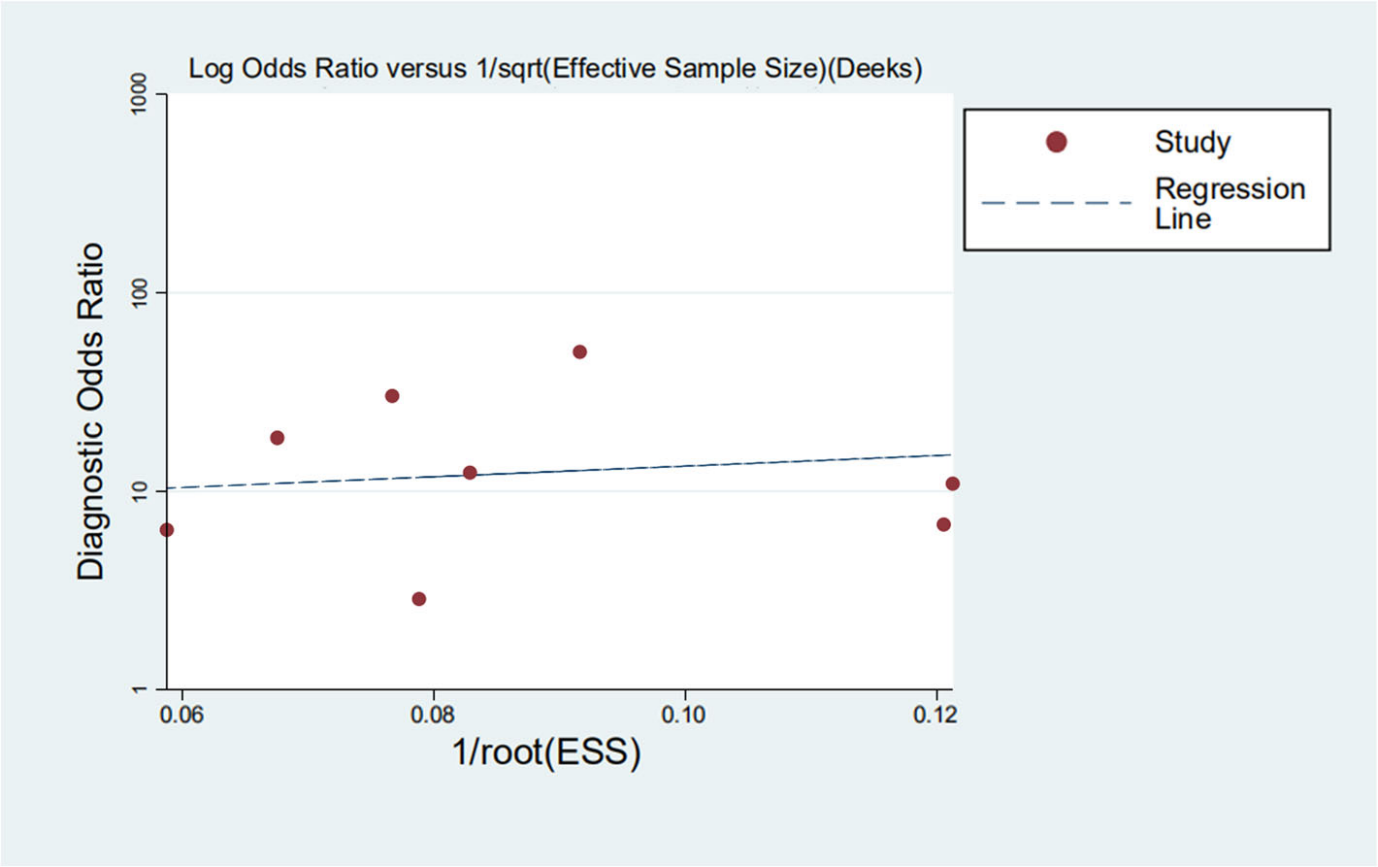
Publication bias from Deeks’ test for Xpert Bladder Cancer in the diagnosis of bladder cancer

Deeks’ funnel plot was used for the evaluation of publication bias in the present study (Fig. 7). No publication bias was found in this research (*P* = 0.755).

### Subgroup analysis

**Fig. 8:**
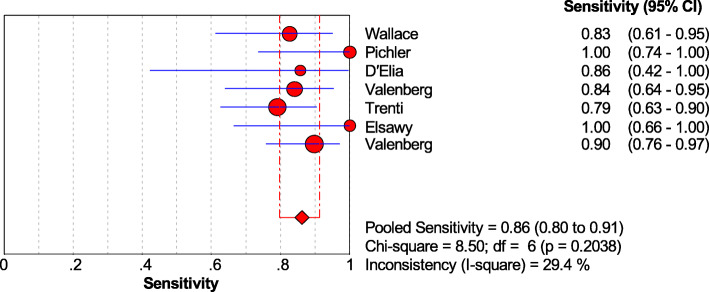
Forest plots of the combined sensitivity of Xpert Bladder Cancer for the diagnosis of high-grade bladder cancer

**Fig. 9:**
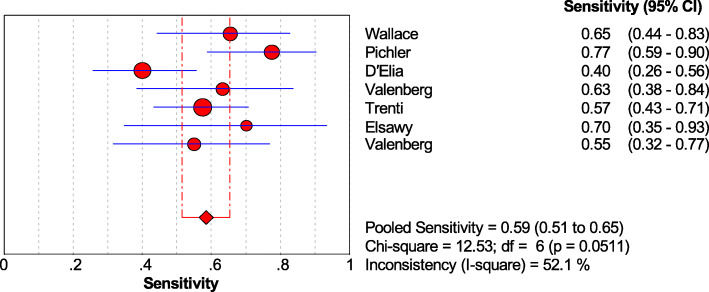
Forest plots of the combined sensitivity of Xpert Bladder Cancer for the diagnosis of low-grade Bladder cancer

Subgroup analysis was performed on the LG and HG of bladder cancer. When combined, the sensitivity of the Xpert BC was 0.86 [95% CI (0.80~0.91)] in HG tumors and 0.59 [95% CI (0.51~0.65)] in LG tumors (Figs. 8 and 9).

## Discussion

This study is probing into the clinical value of Xpert BC in detecting bladder cancer. Eight articles were included finally. The SROC curve is located at the upper left corner, and the value of AUC was 0.8407, indicating that Xpert presents a high accuracy in the diagnosis of bladder cancer. Meanwhile, the deviation value of the included articles was also evaluated by drawing Deeks’ funnel plot. It is generally believed that when *P >* 0.05, it can prove that no publication bias exists in the included literature. The *P* value of our funnel plot is 0.755, which speaks well for no evidence that there is publication bias. The results of heterogeneity analysis show that there is still low heterogeneity, which might be produced for the small number of articles, different research environments, and different LDA settings. In order to further understand the performance of Xpert BC in different degrees of bladder cancer, we classified the sample data of different degrees of malignant transformation. The results show that Xpert BC performs better in the diagnosis of HG than LG bladder cancer.

The sensitivity, specificity, PLR, and NLR of Xpert BC were 0.71, 0.81, 3.74, and 0.34, respectively. And the sensitivity of Xpert BC to HG and LG tumors was 0.86 and 0.59, respectively. Currently, cystoscopy combined with cytology is the gold standard for the diagnosis of bladder cancer. Previous studies showed that the median sensitivity and specificity of cytology for bladder cancer were 35% and 94%, respectively [24]. Compared with cytology for bladder cancer, Xpert BC has higher sensitivity, which ensures the timely detection of recurrence of bladder cancer. In addition, the sensitivity of LG tumor was 0.59, which may lead to the missing of some potential LG cancer patients. The high specificity of Xpert BC prevents the over-treatment of false-positive patients and reduces the waste of medical resources. When the PLR of a diagnostic technique is greater than 10 and NLR is less than 0.1, it indicates that the diagnostic technique is suitable for the diagnosis and exclusion of certain diseases. The PLR and NLR of Xpert BC were only 3.74 and 0.34, respectively. It is suggested that Xpert BC is suitable for further follow-up of patients with bladder cancer, but cystoscopy is still needed for diagnosis and analysis.

Xpert BC is highly valued in monitoring bladder cancer. Although cystoscopy and the cytological test are still adopted as the gold standard to monitor the recurrence of bladder cancer, each of the two methods has its own limitations: cystoscopy is an invasive detection method, which causes uncomfortable feelings to the examinees as well as damage that may induce secondary infection, and as for the cytological test, the sensitivity of the results is not high, and the results are greatly affected by the subjective interpretation of the tester, which deprives the cytological test of authoritative objectivity [25, 26].

Naturally, there are certain limitations to our research: the results show that there is still some heterogeneity in the research, which may be related to the limited literature, different research environments, and different LDA settings. Due to the small number of literature, the grouping of research articles is not the same, which limits the results of using the grouping method to analyze the research content. The difference of LDA values in the included articles, especially in the study of Pichler et al (LDA between − 20 and 20) [5], may affect the interpretation of positive results, thus affecting the heterogeneity value. More studies in the future will contribute to subgroup analysis and heterogeneity resolution.

## Conclusion

In summary, Xpert BC demonstrates high accuracy and specificity in monitoring bladder cancer, compared with cystoscopy tests. More researches are still needed to further confirm our conclusion.

## Supplementary Information

**Additional file 1.** Flow diagram of study selection process

## Data Availability

All data analyzed during this study are included in this published article.

## Abbreviations

BC: Bladder cancer
NMIBC: Non-muscle-invasive bladder cancer
HG: High-grade
LG: Low-grade
TUR: Transurethral resection
Xpert BC: Xpert Bladder Cancer
LDA: Linear discriminate analysis
Ct: Cycle threshold
MeSH: Medical Subject Headings
QUADAS-2: Quality Assessment Standards for Diagnostic Accuracy Studies
PLR: Positive likelihood ratio
NLR: Negative likelihood ratio
DOR: Diagnostic odds ratio
SROC: Summary of receiver operating characteristic curves
AUC: Area under the curve
