# Supplementary material for: Pooled analysis of Xpert Bladder Cancer based on the 5 mRNAs for rapid diagnosis of bladder carcinoma
Source: World J Surg Oncol. 2021 Feb 9;19:42. doi: 10.1186/s12957-021-02154-0 (PMC7874628; doi:10.1186/s12957-021-02154-0)
Supplement: Supplementary file 1 — Additional file 1. Flow diagram of study selection process [file 12957_2021_2154_MOESM1_ESM.docx]

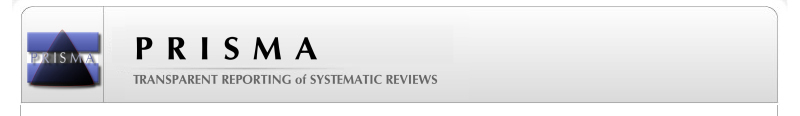
PRISMA 2009 Flow Diagram

Studies included in quantitative synthesis (meta-analysis)
(n=8)

Studies included in qualitative synthesis
(n=8)

Full-text articles

assessed

for eligibility

Records screened
(n=21)

Records after duplicates removed
(n=33)

## Identification

## Eligibility

## Included

## Screening

Additional records identified through other sources
(n=0)

Pubmed (11),

Embase (37),

Cochrane Library (1)

Web of Science (20)

(n=69)

7 articles excluded after reading full text

Reasons for exclusion:

Conference abstract(13)

12 excluded by screening title/abstract
